# Citizens and the public perception of lobbying: do regulation and trust in political institutions make a difference?

**DOI:** 10.1057/s41309-025-00237-x

**Published:** 2025-03-05

**Authors:** Alberto Bitonti, Giulia Mugellini, Claudia Mariotti, Mary Francoli, Jean-Patrick Villeneuve

**Affiliations:** 1 Università della Svizzera italiana (USI), Via Giuseppe Buffi 13, 6900 Lugano, Switzerland; 2 Università degli Studi di Roma Tre, Via Gabriello Chiabrera 199, 00145 Rome, Italy; 3 Carleton University, 1125 Colonel By Drive, Ottawa, ON K1S 5B6 Canada

**Keywords:** Lobbying, Citizens’ perception of lobbying, Public perception of lobbying, Lobbying regulation, Trust

## Abstract

In political studies, lobbying is portrayed as a vital process of political participation, contributing information, policy capacities, and political capital to policymaking, but also as a potential source of representation biases, undue influence, and policy capture. Given such Janus-faced nature of lobbying within democracy, the primary aim of this article is to investigate which perception prevails among citizens empirically. By analysing the primary data of two surveys of 4000 Canadian and 1600 Swiss citizens, it investigates the public perception of lobbying across countries with contrasting institutional and regulatory frameworks and different levels of trust in political institutions. Results show that citizens’ perception of lobbying differs in the two contexts, being predominantly negative in Switzerland and positive in Canada. Additionally, Swiss citizens are significantly more likely than Canadians to view lobbying as inadequately regulated. Both trust in political institutions and the perception that lobbying is properly regulated have a significant and positive impact on the perception of lobbying. Interestingly, despite Switzerland’s higher levels of trust in political institutions than Canada, this trust does not translate into a more positive perception of lobbying, suggesting that robust regulations may play a more decisive role than institutional trust in shaping public perceptions.

## Introduction

If democracy were a theatre production, what part would you assign to lobbyists? Would they more likely be the villains in a story of dark influences or the heroes in a tale of sound policymaking? If you think it depends, you are right. In fact, on the one hand, lobbying by interest groups can be considered a vital component of political participation, fostering collective agency and ensuring that the voices of the governed resonate within the corridors of power, more explicitly representing a channel to improve the flow of information, policy capacities and political capital into actual policymaking processes (Baumgartner and Leech 1998; Bouwen 2002; Deardorff and Hall 2006; Albareda et al. 2023). On the other hand, lobbying by interest groups can be a source of representation biases, undue influence, and policy capture (Schattschneider 1960; Carpenter and Moss 2013; Lowery et al. 2015; OECD 2017), where the more resourceful players might wield more power than what the “force of the better argument” would give them on a purely “rational” level (Habermas 1996; Shapiro 1999).

It is probably because of this latter view that lobbying as an activity and as a profession is often tainted by a bad reputation, with conceptual overlaps sometimes made not only with undue influence but even corruption (Campos and Giovannoni 2007; Holyoke 2015; Goldberg 2018; Ron and Singer 2020). Such negative connotations can be frequently found not only in fictional representations of movies and TV series, but also in the non-fictional representations provided by news reporters, pundits, and political actors. Often, even lobbyists themselves strategically exploit the rhetorical trope of the lousy lobby versus the public interest against each other (Bitonti 2020; Raknes and Ihlen 2020), resulting in an interesting disassociation of roles and self-perceptions.

Given such Janus-faced, dual nature of lobbying within democracy, various research questions are relevant to be tackled on the empirical level. First, which of the two faces do citizens mainly perceive? In other words, what is the public perception of lobbying? Having solid data and shedding light on this aspect is crucial to better understand what the public attitude towards a key dynamic of modern democracies is, just as it happens with the public perception of political actors and institutions such as political parties or parliaments. While some studies have investigated the perception of lobbying conveyed by journalists, policymakers, and lobbying professionals (McGrath 2013; Davidson and Rowe 2016; von den Driesch and van der Wurff 2016; Helgesson and Falasca 2017; McGrath 2018; Koch and Schulz-Knappe 2021), little research has been produced so far focussing on citizens (Larsson 2007; Bitonti and Harris 2017). Generally, the perception of lobbying by citizens is assumed to be consonant with the representations provided by media and policymakers and, therefore, predominantly negative. The first aim of this article is to empirically test this assumption, directly looking at citizens’ perceptions.

Second, does lobbying regulation make a difference, impacting public perceptions? The literature on lobbying regulation usually recalls the argument that regulating lobbying (well) leads to more public integrity, transparency, accountability, higher trust, and better policies (Davidson 2017; Chari et al. 2019; Porumbescu et al. 2022; Bitonti and Mariotti 2023). Does this also entail a better view of lobbying itself? Can we find a more positive perception of lobbying in countries with more robust regulations? The second aim of our research is to provide an empirical answer to this question.

Third, what is the relationship between trust in political institutions and the public perception of lobbying^1^? While the issue of trust in political institutions is widely studied and explored in various international surveys (Haerpfer et al. 2022; OECD 2022; Winsvold et al. 2024), little research has been produced connecting this issue with lobbying regulation (Kanol 2018; Crepaz and Arikan 2024). However, to our knowledge, no research has specifically explored the relationship between trust in political institutions and citizens’ perception of lobbying. Our research aims to fill this gap.

We tackle the three research questions by analysing the results of two national surveys submitted to representative samples of the citizens of Canada and Switzerland (*N* = 4000 + 1600). By focussing on these two national cases, we develop a comparative analysis that not only provides new empirical data on the public perception of lobbying in these contexts but also tests some theoretical expectations connecting citizens’ perception of lobbying (as a dependent variable) with different lobbying regulatory frameworks and levels of trust in political institutions (as independent variables).

The article is structured as follows. The next section explores the literature on the role and perception of lobbying within democratic systems. A brief overview of the lobbying regulatory context in Canada (more developed and object of specific legislation) and Switzerland (lacking specific legislation) follows. Then, the research design and methodology are elaborated, followed by a discussion of our findings and theoretical interpretations. The article concludes with a look at the study's limitations and potential directions for future research.

## Lobbying, democracy, and perceptions

For decades, political scientists have attempted to formulate macro-theories concerning the balance of interests existing in numerous countries at various times, providing diverse analyses under the theoretical perspectives of pluralism, neo-corporatism, and neo-pluralism (Truman 1951; Schattschneider 1960; Lowi 1979; Lindblom 1980; Gilens and Page 2014). Different arguments are used to describe the theoretical relationship between lobbying and democracy, deeming the former either as a functional expression of or as a threat to the latter, often grounding this judgement precisely on the macro-perspectives mentioned above.

The “positive” view (lobbying as a functional expression of democracy) is based on various arguments. Some highlight how lobbying is a form of collective action and political participation, shielded by the liberal-democratic ideals of pluralism, freedom of speech, and the right to petition the government (Truman 1951; Milbrath 1963; Holyoke 2015). Others stress the “exchange logic” that drives the relationship between interest groups supplying information and demanding access and policymakers demanding information and supplying access (Bouwen 2002). It is worth noting that such information may take the form of technical expertise or concern the policy preferences of the public or of specific groups (Baumgartner and Leech 1998; Flöthe 2019). In this regard, lobbying would even constitute a form of “legislative subsidy” for policymakers (Deardorff and Hall 2006), in need of various policy capacities offered by interest groups (Albareda et al. 2023). The key vision here is ‘the competition of ideas [that] occurs as a contest among partisans’ (Lindblom 1980, p. 35).

The negative view (lobbying as a threat to democratic ideals) may be developed, instead, from three core arguments. The first argument can be traced back to Plato’s technocratic views, fundamentally opposing democracy and pluralism as something good. This is the case of all absolutist doctrines defending substantive visions of the public interest, where ‘the greater good cannot be “distorted” by partial (i.e. particular, sectional, or special) interests, seen as biased expressions of factions, lacking the full vision, and characterized by ignorance, evilness, or selfishness’ (Bitonti 2020, p. 5). The second argument is based not on a normative denial of democracy or pluralism, but less fundamentally on the concern that existing inequalities in society make the pluralist competition of ideas more an ideal than a reality (Schattschneider 1960; Shapiro 1999). This means that inequalities between different interest groups in terms of resources, access, and concrete possibilities to influence policymaking make lobbying itself problematic in democratic terms. In fact, it would represent a potential source of representation bias, where some interests “count” disproportionately more than others (Lowery et al. 2015; Goldberg 2018), or even policy capture, where the regulators end up following the directions of the regulated (Carpenter and Moss 2013; OECD 2017). The third argument focuses on the procedural requirements of democracy, and “attacks” lobbying as a practice that may take place in unethical and non-transparent ways (McGrath 2005, 2018; Holyoke 2015), harming the principle of open and fair democratic deliberations (Habermas 1996; Bitonti and Mariotti 2023).

Given this theoretical background, various research efforts have been made to empirically assess how lobbying and its role in democracy are perceived. The bulk of such research has so far focussed on media outlets, policymakers, and lobbying professionals themselves. For instance, Helgesson and Falasca (2017) explored how Swedish media framed the role and practice of lobbying between 2006 and 2016, evidencing a common and persistent negative frame in general news media. McGrath (2018) investigated how lobbying was depicted in British newspapers and in policymakers’ discourses in the British Parliament between 1800 and 1950, also finding a predominance of negative tones (53.6% of the cases) in front of a tiny minority of positive representations (3.5% of the cases). Policymakers’ rather negative stance on lobbyists and lobbies is not confined to the past though. Presidential candidates in the US, from both the Democratic and the Republican sides, have used the trope of “draining the swamp” and limiting the influence of lobbyists in Washington DC multiple times (McGrath 2013).

Various scholars have further investigated how lobbyists themselves conceive their role in democracy. For instance, Davidson and Rowe (2016) surveyed both British public affairs practitioners and various British and American opinion makers (Members of Parliament, journalists, political professionals), evidencing how on the one hand lobbying plays an important role in democracy, but on the other a poor image of the profession tends to persist for the public, especially because of misperceptions of their job, association of ideas with political scandals, and lack of transparency. In another study surveying Dutch lobbyists, Von den Driesch and Van der Wurff (2016) highlight how the majority conceive their roles as advocates for their organizations, with many also emphasizing their role as experts and mediators (improving policymaking in terms of technical knowledge and balance with other stakeholders’ interests). Similar orientations also emerge in Koch and Schulz-Knappe’s study (2021) on German lobbyists and in Helgesson’s study (2023) on Swedish public affairs consultants.

When it comes to citizens’ views, various studies have analysed public relations (PR) professionals’ reputation. For instance, Callison (2001) experimentally tested the perception of the credibility of PR professionals among American teachers (representatives of average news readers), and Larsson (2007) relied on national surveys of Swedish citizens. To the best of our knowledge, to date, no study has specifically addressed citizens’ perception of lobbying and lobbyists, with the exception of Bitonti and Harris (2017), who collected experts’ views concerning the public perception of lobbying in the European Union member states.

Overall, existing literature stresses how lobbying (as well as public relations more generally) does suffer from a bad reputation among most citizens, at least in the few contexts that have been studied. Our research aims not only to produce new evidence, but also to take advantage of a comparative research design to engage in a more fine-grained and nuanced analysis of this issue.

## Lobbying regulation in Canada and Switzerland

If openness and fairness in the “influence game” constitute the basic conditions of democratic dynamics shaping the views on lobbying as discussed above, one can easily understand the importance of lobbying regulation in this regard. In fact, lobbying rules can substantially affect the interactions and the dynamics of power involved in the relationship between interest groups and policymakers (Davidson 2017; Chari et al. 2019; Bitonti and Mariotti 2023; Laboutková and Vymětal 2023). It is reasonable to assume then that different lobbying regulatory frameworks will produce different lobbying environments. That is why this section provides a short overview of the lobbying regulatory frameworks in the two contexts under analysis: Canada and Switzerland.

The most evident difference between Canada and Switzerland is that while Canada has (since 1989, with various later amendments) legislation specifically dedicated to regulating lobbying, Switzerland has none^2^ (Chari et al. 2019).

Canada employs a disclosure-based system, requiring all lobbyists to register and report their activities in a federal register (with other sub-national administrations also regulating lobbying through similar measures at regional or even municipal level). Lobbyists are defined as individuals who, for payment, on behalf of any person or organization (both as consultants or in-house professionals), seek to communicate with public office holders trying to influence their decisions or arrange a meeting between them and any other person (Section 5 of the Lobbying Act of 2008). Such a definition is mirrored by the concept of designated public office holders (DPOH), including executive branch officers as well as the members of Parliament (both House of Commons and Senate) and other federal officials. Re-registration and filing of information by lobbyists occur, monthly, if contact is made with DPOH. The information provided to the register covers not only the lobbyists’ and their organization’s identities, but also various details on the aim of the lobbying activity, its object, and the communication techniques used (including grassroots lobbying campaigns). Other provisions of the Canadian law regard cooling-off periods (the time a former public office holder needs to wait before being allowed to become a lobbyist: five years in most cases), the implementation of the law (the establishment of the Office of the Commissioner of Lobbying, the lobbyists’ Code of Conduct), as well as its enforcement (contraventions and punishments). Overall, the rationale of Canadian legislation on lobbying is the pursuit of transparency, rooted in the citizens’ right to know who influences whom on what decisions.

As mentioned, Switzerland does not have any similar piece of legislation specifically dedicated to lobbying (Ammann 2024). That is why, when Chari et al. rank the robustness of lobbying laws using the Center for Public Integrity Index (Chari et al. 2019, p. 183), Canada scores pretty well (50 points out of 100, only second to the USA, scoring 62), and Switzerland does not even appear on the list. However, following a wider definition of lobbying regulation, not limited to lobbying laws but including all legislative and non-legislative norms affecting lobbying activities (Bitonti and Mariotti 2023, p. 147), more details may be added to this picture as both countries have a range of tools to guard against undue influence of interests groups and to ensure government accountability. For instance, even if Switzerland does not have a specific piece of legislation dedicated to regulating lobbying, the Federal Council (the Swiss executive branch) and the Federal Assembly (the Parliament, composed of two chambers: the National Council and the Council of States) widely use different tools such as (respectively) consultations and hearings with interest groups when preparing legislative dossiers and bills, with a very meaningful role also played by popular initiatives and referenda (Sciarini et al. 2015; Christiansen et al. 2018; Eichenberger 2020). The Swiss Parliament can be also deemed quite transparent and accountable as for potential conflicts of interest of its members, with the full list of memberships to associations and employments for each member of the Parliament published annually on the institution’s website (under the name of Register of Interests), according to article 11 of the Parliament Law of 2002. On the other hand, the typically Swiss militia principle results in many members of Parliament having side occupations and employments, making the picture more complex (Wiesli 2003; Ammann 2024). The Federal Legislation on Political Rights of 1976 and the Ordinance on Transparency in Political Funding of 2022 should also be mentioned, as they regulate political financing by establishing some bans (anonymous donations above 15,000 francs and foreign donations) and mandatory disclosures (for donations above 15,000 francs).

It should be noted that in addition to formalized lobbying legislation, Canada also has a wider regulatory and consultative toolbox similar to the Swiss context described above. Issue experts, including representatives from interest groups, are often invited to give input on draft policy and legislation and parliamentary committees regularly invite them to testify when the committees are engaging in special studies or legislative review (transcripts of such testimony can be found at parl.ca). In addition to this, the government also has a long history of running public consultations on a wide range of policy issues (see https://www.canada.ca/en/government/system/consultations/consultingcanadians.html). At the time of writing, for example, 926 consultations held between 2016 and 2024 were listed.

As is the case in Switzerland, Canada also has a long history of initiatives aimed at mitigating conflict of interest in Parliament. Rules predate the 1900s and are currently reflected in the Conflict of Interest Act that came into force in 2007 (https://ciec-ccie.parl.gc.ca/en/About-APropos/Pages/History-Histoire.aspx). The Act is expressly oriented at preventing conflicts of interest between parliamentarians and private interests. In the public services, the Public Servants Disclosure Protection Act, which also came into force in 2007, sets out a mechanism for allowing public servants to report potential wrongdoings and conflicts in the public service. Both the Conflict of Interest Act and the Public Servants Disclosure Protection Act were part of a suite of changes made under the Federal Accountability Act, an omnibus bill that made changes to approximately 50 other pieces of legislation with the aim of improving government accountability and transparency. This also includes legislation on political financing.

In conclusion, both Canada and Switzerland display a variegated regulatory framework. Yet, while Canada has a relatively long tradition of tackling the issue of lobbying regulation with legislative measures and administrative offices—thus displaying a higher level of robustness—in Switzerland lobbying is only regulated indirectly and in other forms. For the scope of the present article, such a difference is sufficient to draw a meaningful comparison in this regard between the citizens’ perceptions of lobbying in the two cases.

## Research design and methodology

To answer the three research questions elaborated earlier in this article, hypotheses from the literature are drawn, and tested through a comparative research design, using Canada and Switzerland as cases of study.

This choice is informed by both theoretical and empirical considerations. From a theoretical standpoint, drawing on the comparative research literature, this study considers elements of both the most similar systems design (MSSD) (Lijphart 1971) and the most different systems design (MDSD) (Przeworski and Teune 1970) to justify the choice of Canada and Switzerland. These two countries provide an ideal comparative framework because they share key structural similarities but also exhibit significant differences that allow for a robust analysis of the determinants of lobbying perceptions. In particular, they share core characteristics that make them comparable, such as being both democratic countries with a federal structure where power is decentralized to regional governments and a strong rule of law. In addition, both countries are economically developed, with stable political systems. These similarities allow to control for broad systemic factors that might otherwise confound the analysis. By focussing on two countries that share democratic and federal structures, it is possible to better isolate and examine the impact of the specific variables of interest (i.e. citizens' perceptions of lobbying, trust in political institutions, and other sociographic factors).

Despite they share democratic and federal frameworks, Canada and Switzerland differ in terms of political systems and lobbying regulations. Canada operates under a Westminster parliamentary system, while Switzerland follows a consociational semi-direct democracy that emphasizes consensus and regular citizen participation (Lijphart 1999; Sciarini et al. 2015). Canada has a more formalized and transparent system of lobbying regulation, with strict disclosure and registration requirements, while Switzerland’s system is less institutionalized and has less stringent transparency requirements (see previous section). These differences provide an opportunity to test how divergent institutional arrangements and regulatory frameworks influence the public perception of lobbying in each context.

The idea is to use MSSD to control for common institutional and democratic structures (i.e. federalism, rule of law, democracy) in both Canada and Switzerland, but then leveraging elements of MDSD to explore how the differences in political system and lobbying regulations impact the public perception of lobbying. This allows to assess how much of the difference in public perceptions can be attributed to the specific institutional differences between the two countries. By controlling for the broader systemic similarities (MSSD), this study can argue that the differences in lobbying regulation and political systems are key determinants of the public perception of lobbying. Meanwhile, by using MDSD logic, it suggests that the hypotheses we are testing are robust across different political systems. Furthermore, by combining MSSD and MDSD, the study allows for both generalization and specificity (Przeworski and Teune 1970) and helps strengthening the external validity of the conclusions. The similarities between the two countries allow to isolate certain variables, while the differences allow to test the robustness of the hypotheses.

The following section introduces the hypotheses and describes the methodology.

### Questions and hypotheses

As explained in the introduction, our first research question reads as follows:

#### RQ1

How do citizens perceive lobbying in its relationship to democracy?

Here our expectation, drawn from previous studies on the perception of lobbying and public relations by media elites, policymakers, and citizens, is that citizens mostly have a negative view of lobbying, considering it a channel of undue influence or even corruption, and thus a danger for democracy. We expect such a negative view to prevail in both contexts under analysis, operationalising it as more than 50% of citizens adopting such a position. So, our first hypothesis reads as follows:

#### H1

Citizens tend to have a negative perception of lobbying.

Our second research question is the following:

#### RQ2

How does lobbying regulation relate to the public perception of lobbying?

Our expectation, drawn from the literature on lobbying regulation, is that a more robust lobbying regulatory framework—such as the Canadian one in comparison to the Swiss one—is associated with a better perception of lobbying among citizens, operationalising this with a higher frequency of positive views and lower frequency of negative ones in Canada in comparison to Switzerland. Another proxy variable is used to approximate the robustness of lobbying regulation, referring to the citizens’ perception of the appropriateness of lobbying regulation in their own country. So, our second hypothesis reads as follows:

#### H2

Citizens’ perception of lobbying is better in countries with more robust lobbying regulations.

Our third research question is the following:

#### RQ3

How does trust in political institutions relate to the public perception of lobbying?

Here, our expectation is that, similarly to what happens with the robustness of lobbying regulations, we find a better perception of lobbying (operationalised again with a higher frequency of positive views and a lower frequency of negative ones) where the level of trust in political institutions is higher, and so where the accountability of political institutions and the “resistance” to undue influences or potential threats from lobbying actions is presumably higher, defusing risks for democratic policymaking. Thus, our third hypothesis reads as follows:

#### H3

Citizens’ perception of lobbying is better in countries with higher levels of trust in political institutions.

### Methodology

The methodology relies on a sample survey of Canadian and Swiss citizens (18 years or older with political rights). The questionnaire of the sample survey is structured to collect data on different phenomena, such as the perception of lobbying and trust in political and public institutions. The structure and content of the questionnaire draw on existing and consolidated surveys, such as the World Values Survey (Haerpfer et al. 2022).

Computer Assisted Web Interviewing (CAWI) was used for administering the questionnaire to the sample of Swiss and Canadian citizens. The choice of this specific data collection method was mainly affected by the need to investigate also potentially sensitive issues. This method belongs to the so-called self-administered category of data collection through which the respondents can compile the survey questionnaire by accessing it via web through a personal username and password. This method is recognized as one of the best to cope with social desirability bias. This concept, introduced by methodologists in the 1950s, refers to the tendency of interviewees to respond taking into account the so-called generalized other, that is the representation that an individual gives to oneself on what is considered appropriate and right within their culture (Goffman 1959). The absence of an interviewer reduces social interaction and might also reduce respondents’ tendency to take into account social norms, especially when introducing implicit measures of a specific phenomenon, as opposed to direct questions, to avoid distorted responses (e.g. Implicit Association Test)(Egloff and Schmukle 2002). However, the literature shows that no data collection method is exempt from potential social desirability bias (Bradburn et al. 1978).

As both Canada and Switzerland are multilingual countries, the questionnaire was translated into four languages: English, French, German, and Italian. A pilot survey was conducted in June 2023 on a sample of 100 Canadian Citizens and 51 Swiss citizens. The questionnaire has been then updated and the final survey was conducted in September 2024.

The surveys were carried out by two well-known polling and marketing research firms (Abacus in Canada and YouGov-CH in Switzerland) with longstanding experience in setting up and conducting complex social research surveys. Cooperation with experienced firms ensured the soundness of the project methodology, the standardization of the data collection method, and access to their panels of individuals.

To test the hypotheses of this study, the data collected through the pilot surveys are analysed through descriptive and inferential statistics. Both bivariate and multivariate analyses are developed, such as cross-tabulations with the analysis of Chi-square of Pearson and its Significance (as a test of a statistical association between two different variables in the cross-tabulations), Cramer V and its Significance, Odds ratio and Confidence Intervals, Phi coefficient and its Significance. Binary and multinomial logistic regressions, together with linear regression are performed for multivariate analyses. This method combines several independent variables to estimate the probability that a specific event will happen (in this case the negative/positive perception of lobbying).

Citizens’ perception of lobbying is the dependent variable of this study, and it is measured through one main question, recalling the idea of lobbying as beneficial or detrimental to democracy (see Table 1). The different shades of perception are operationalized through a Likert scale (from 1 to 7). Likert scales allow for the quantitative measurement of attitudes and perceptions and are useful to provide a more nuanced understanding of respondents' views on a given phenomenon (Corbetta 2003). The Likert scale has been recoded to ease the interpretation of cross-tabulations and to ensure the reliability of the multinomial logistic regression model (see Table 7 in the Appendix). However, the original Likert scale has been used as dependent variable in a linear regression model which represents a sensitivity analysis to confirm the results of the logistic regression while accounting for the full data complexities (see Table 11 in the Appendix).

**Table 1:** Perception of lobbying by country: aggregated results and % in Canada and Switzerland

	Canada		Switzerland		Total	
	*N*	%	*N*	%	*N*	%
Lobbying is a danger for democracy (Negative)	1133	28.7	960	58.1	2093	37.4
Neither a danger nor beneficial (Neutral)	1265	32.1	491	29.7	1756	31.4
Lobbying is beneficial for democracy (Positive)	1548	39.2	200	12.1	1748	31.2
Valid total	3946	100	1651	100	5597	100

*X*^2^ (1, *N* = 5597) = 545.698, *p* ≤ 0.001

The robustness of lobbying regulation, one of the independent variables, is considered as a macro dichotomic variable overlapping with the country case (we assume that in Canada, the regulation is more robust than in Switzerland). In addition, a binary variable on citizens’ perceived appropriateness of lobbying regulations is also used to measure the concept of robustness of lobbying regulation (see Table 8 in the Appendix). The second independent variable concerns citizens’ trust in political institutions. This concept is measured as the arithmetic mean of six different variables on the level of trust in different political institutions (i.e. the Parliament/General Assembly, local representatives, provincial/cantonal representatives, political parties, the party in power/Federal Council, the Prime Minister/President) (see Table 9 in the Appendix). A reliability test, with Cronbach’s Alpha has been conducted to ensure the internal consistency of these six variables in measuring the same underlying construct in political institutions (see Table 11 in the Appendix). The relationship between the dependent and independent variables has been controlled for different factors indicated as potentially relevant confounders by the literature: trust in executive power and public institutions, political orientation, gender, age, and level of education.

The sample of the survey has been drawn to be representative of the population at cantonal level for Switzerland, and provincial level for Canada by gender and age. The survey includes responses from 4000 Canadian citizens and 1600 Swiss citizens. Table 5 in the Appendix describes the main sociographic characteristics of both samples.

## Results

The analysis results partially confirm hypothesis H1, claiming that, in general, citizens tend to have a negative perception of lobbying. Indeed, looking at the sample as a whole (see Table 1), the majority (37.4%) of respondents, even if slight, position themselves on the negative side of lobbying perception. A “neutral” perception of lobbying, perceived as neither a danger nor beneficial for democracy, is as frequent as a positive one (around 31%).

However, when analysing the two countries separately, results show significantly different perceptions of lobbying among Canadians and Swiss citizens. Swiss respondents are much more likely to have a negative perception of lobbying (58.1%) compared to Canadians (28.7%). Canadians are significantly more likely to have a positive perception of lobbying (39.2%), whereas only 12.1% of Swiss respondents share this view. The neutral category is relatively similar between the two countries, with about a third of respondents from both countries expressing neutrality. The differences in the distribution of perceptions of lobbying between Canada and Switzerland are statistically significant. This finding supports our second hypothesis (H2), that in a country with more robust lobbying regulations (i.e. Canada) citizens have a better perception of lobbying in comparison to a country with less robust or no regulation (i.e. Switzerland).

In addition to this, it appears also a significant and strong relationship between perceptions of whether lobbying is properly regulated in the country and the respondents' perceptions of lobbying itself. Those who think lobbying is properly regulated tend to have a more positive view of lobbying (see Table 2 below).

**Table 2:** Perception of lobbying by perception of appropriate lobbying regulations: aggregated results and valid % in Canada and Switzerland

Appropriateness of lobbying regulation	Lobbying is properly regulated		Lobbying is NOT properly regulated		Don’t know		Total	
Lobbying perception	N	%	N	%	N	%	N	%
Lobbying is a danger for democracy (Negative)	209	19.2	1420	58.7	463	22.2	1629	46.4
Neither a danger nor beneficial (Neutral)	301	27.6	489	20.2	966	46.3	790	22.5
Lobbying is beneficial for democracy (Positive)	580	53.2	512	21.1	656	31.5	1092	31.1
Valid Total	1090	100	2421	100	2085	100	3511	100

*X*^2^ (1, *N* = 3511) = 519.285, *p* ≤ 0.001

The negative perception of lobbying is highest among those who think lobbying is not properly regulated (58.7%). Among those who think lobbying is properly regulated, the negative perception is much lower (19.2%), as well as among those who answered "I don’t know," (22.2%). The “neutral” perception is quite dominant in the "I don’t know" group (46.3%), suggesting that uncertainty about regulation correlates strongly with neutrality in lobbying perception. For a positive perception of lobbying, the highest proportion (53.2%) is found among those who think lobbying is properly regulated, but a significant portion (31.5%) of the “I don’t know” group also views lobbying positively.

Moving to our third hypothesis (H3), we expected to find a better perception of lobbying where trust in political institutions is higher.

In line with existing research (Haerpfer et al. 2022), our analysis confirms that, in general, trust in political institutions is a significant predictor of perception of lobbying, with high trust strongly associated with positive views of lobbying (see Table 3). Across both countries, the proportion of respondents with a negative perception of lobbying decreases as trust increases, from 39.9% in the low-trust group to 32.3% in the high-trust group. Similarly, positive perception of lobbying increases from 27.0% in the low-trust group to 44.4% in the high-trust group.

**Table 3:** Perception of lobbying and trust in political institutions: crosstabulation with chi-square analysis

Country				Trust Political Institutions			
				Low	Medium	High	Total
Canada	Lobbying perception	Negative	N	929	60	125	1114
			% within Trust	32.4%	27.0%	16.8%	29,1%
		Neutral	N	986	56	149	1191
			% within Trust	34.4%	25.2%	20.0%	31,1%
		Positive	N	952	106	470	1528
			% within Trust	33.2%	47.7%	63.2%	39,9%
	Total	N	2867	222	744	3833	
		% within Trust	100%	100%	100%	100%	
Switzerland	Lobbying perception	Negative	N	557	75	316	948
			% within Trust	64.7%	63.0%	51.0%	59,3%
		Neutral	N	250	34	168	452
			% within Trust	29.0%	28.6%	27.1%	28,3%
		Positive	N	54	10	136	200
			% within Trust	6.3%	8.4%	21.9%	12,5%
	Total	N	861	119	620	1600	
		% within Trust	100%	100%	100%	100%	
Total	Lobbying perception	Negative	N	1486	135	441	2062
			% within Trust	39.9%	39.6%	32.3%	38,0%
		Neutral	N	1236	90	317	1643
			% within Trust	33.2%	26.4%	23.2%	30,2%
		Positive	N	1006	116	606	1728
			% within Trust	27.0%	34.0%	44.4%	31,8%
	Total	N	3728	341	1364	5433	
		% within Trust	100%	100%	100%	100%	

*X*^2^ for Canada (1, *N* = 3833) = 228.370, *p* ≤ 0.001, *X*^2^ for Switzerland (1, *N* = 1600) = 84.728, *p* ≤ 0.001, *X*^2^ Total (1, *N* = 5433) = 145.502, *p* ≤ 0.001

Looking at the two countries separately also yields interesting insights. Trust in political institutions is higher in Switzerland than in Canada. A significantly higher proportion of respondents in Switzerland have high trust in political institutions compared to Canada (38.8% vs 19.4%). A higher proportion of respondents in Canada have low trust compared to Switzerland (74.8% vs 53.8%). This suggests that Swiss respondents have more confidence in their political institutions than Canadians. However, in Switzerland, respondents with low trust are particularly likely to have negative perceptions of lobbying. Indeed, a much higher proportion of Swiss respondents with low trust in political institutions have a negative perception of lobbying (64.7%), compared to Canadians (32.4%). Interestingly, when considering the level of trust for the six political institutions separately, in Switzerland, it is generally higher than in Canada, with approximately 40% of Swiss respondents expressing high trust compared to around 20% of Canadian respondents. However, an exception to this trend is trust in political parties, which remains consistently lower, at about 20% in both countries.

In Canada, high trust in political institutions is more strongly associated with positive views of lobbying, suggesting that trust in institutions may play a more influential role in shaping favourable views of lobbying. In particular, 63.2% of Canadians with high trust in political institutions view lobbying positively, while in Switzerland only 21.9% of respondents with high trust have a positive perception.

Cross-tabulations have been performed for all the different six political institutions separately, namely the executive power, provincial/cantonal representatives, local representatives, political parties, and the prime minister/president. The relationship between trust in these institutions and the perception of lobbying was always significant and moderately strong in both countries (see Appendix).

A multinomial regression was performed to further investigate the relationship between the perception of lobbying and the level of trust in political institutions, controlling for the relevant confounders (appropriateness of lobbying regulation, political orientation, gender, age, and level of education). Its results are reported in Table 4 as follows:

**Table 4:** Perception of lobbying and trust in political institutions: multinomial regression

*Model fitting information*	*Log-likelihood -2*		*Chi-Square*	*df*	***p-value*** ≤
Model intercept only	2908.767		-	-	-
Final model	1121.057		1787.710	16	<.0001
*Pseudo R-square*					
Cox and Snell R-squared	0.282				
Nagelkerke R-squared	0.318				
% of zero cells	8.6%				
Dependent variable	Perception of lobbying (Negative; Neutral; Positive) (positive is the reference category)				
Independent variables in the equation	B	SE	Wald	-	Exp(B)
Intercept (Negative)	0.838	0.132	40.168	< 0.001	
Country (Canada vs Switzerland)	−2.246	0.102	480.63	< 0.001	0.106
Trust in political institutions (Low vs High)	1.031	0.095	116.888	< 0.001	2.804
Trust in political institutions (Medium vs High)	0.728	0.167	19.017	< 0.001	2.071
Political orientation (Right vs Left)	−0.587	0.078	55.961	< 0.001	0.556
Gender (Male vs Female)	0.312	0.077	16.491	< 0.001	1.366
University (No vs Yes)	−0.111	0.079	1.967	0.161	0.895
Lobbying properly regulated (No vs Don't Know)	1.158	0.087	175.833	< 0.001	3.183
Lobbying properly regulated (Yes vs Don't Know)	−0.834	0.115	52.246	< 0.001	0.434
Intercept (Neutral)	0.87	0.131	43.779	< 0.001	
Country (Canada vs Switzerland)	−1.536	0.104	218.667	< 0.001	0.215
Trust (Low vs High)	0.962	0.092	108.782	< 0.001	2.618
Trust (Medium vs High)	0.559	0.166	11.308	< 0.001	1.749
Political Orientation (Not Left vs Left)	−0.382	0.076	25.01	< 0.001	0.682
Gender (Male vs Female)	−0.248	0.075	10.886	< 0.001	0.781
University (No vs Yes)	0.326	0.08	16.535	< 0.001	1.386
Lobbying Regulated (No vs Don't Know)	−0.37	0.087	18.031	< 0.001	0.691
Lobbying Regulated (Yes vs Don't Know)	−0.838	0.098	72.808	< 0.001	0.433

After testing several combinations of regressors, this final model was selected as the best trade-off among predictors’ significance (Likelihood Ratio Tests for each predictor showed highly significant results, *p* < 0.001), model fit/explained variance (Nagelkerke: 0.318, or 32%), stability (8.6% zero cells), and interpretability of results. Additional details on model selection are provided in the Appendix

The overall model performance and stability are good and in line with standards for robust results in social and political science (Ozili 2023). In particular, the Nagelkerke *R*^2^ is approximately 0.318 (or 31.8%), showing a solid level of explained variance for multinomial logistic regression in social sciences, especially when perceptions are studied (ibidem). This suggests that the model's predictors account for about 32% of the variability in lobbying perceptions (the dependent variable). The large and significant chi-square improvement from the intercept-only model to the final model (1787.710, *p* < 0.001) indicates that the predictors significantly improve the model’s fit. The model’s stability (low zero-cell percentage, 8.6%^3^) and reasonable *R*^2^ values (Cox, Snell, and Nagelkerke) further suggest the model is robust.

In terms of predictors, results show that the variable “Country” has a significant negative effect on the likelihood of perceiving lobbying as a danger to democracy. This suggests that, holding all the other variables constant, Swiss citizens are more likely to perceive lobbying negatively, or in a neutral way, in line with our second hypothesis. In particular, respondents from Canada are 0.106 times (89.4%) less likely to have a negative perception of lobbying compared to those from Switzerland (*p* < 0.001). Similarly, Canadians are 0.215 times less likely to have a neutral perception compared to Swiss respondents (*p* < 0.001).

The second hypothesis is further confirmed by the results linked to the variable “Lobbying properly regulated”. Indeed, respondents who believe lobbying is not properly regulated are substantially (3.183 times) more likely to have a negative perception of lobbying (*p* < 0.001) and 0.691 times (30.9%) less likely to have a neutral perception (*p* < 0.001). On the other side, respondents who believe lobbying is properly regulated are much less likely to hold either a negative (0.434 times or 56.6% less likely) or neutral (0.433 times or 56.7% less likely) perception (*p* < 0.001). Belief in proper regulation of lobbying greatly reduces the likelihood of having a negative or neutral perception of lobbying. In other words, people who think lobbying is properly regulated are far more likely to view lobbying in a positive light. This highlights the impact of perceived regulatory adequacy on attitudes towards lobbying, with the perception of inadequate regulation driving negative views.

The third hypothesis is also confirmed as trust in political institutions plays a crucial role in shaping lobbying perceptions. Respondents with lower levels of trust are significantly more likely to have negative or neutral perceptions of lobbying than those with higher trust. This effect is stronger in the “Low trust” category, indicating that respondents with lower trust in political institutions are 2.804 times more likely to have a negative perception of lobbying than those with high trust (*p* < 0.001). They are also 2.618 times more likely to have a neutral perception than those with high trust (*p* < 0.001). Similarly, respondents with medium trust are 2.071 times more likely to have a negative perception of lobbying (*p* < 0.001) and 1.749 times more likely to have a neutral perception (*p* < 0.001) compared to those with high trust.

The other variables included in the regression, the political orientation of respondents, their gender, and level of education, are also significantly affecting the perception of lobbying. In particular, respondents who are left-oriented are more likely to view lobbying negatively or neutrally, and men are 1.366 times more likely to have a negative perception compared to women (*p* < 0.001). However, women are more likely to have a neutral perception. Individuals without a university education are significantly more likely to hold a neutral perception of lobbying than a positive one. However, their likelihood of holding a negative view compared to a positive one is not significantly different. This may imply that those with lower education levels may have a less critical but also less favourable stance on lobbying.

## Discussion

This study aimed to empirically investigate which of the two ‘faces’ of lobbying prevails in the public perception of citizens, drawing a comparative analysis of the cases of Canada and Switzerland. Significantly different perceptions of lobbying emerged, with Canadians having a much more positive attitude towards lobbying than Swiss citizens. This difference may be explained by returning to existing literature. First, in line with what the literature on lobbying regulation has been assuming for years, a more robust lobbying regulatory framework (such as the Canadian one) is associated with a better perception of lobbying. More robust regulations result in more open lobbying environments and more transparent interactions between interest groups and policymakers. This makes lobbying less problematic in democratic terms (Chari et al. 2019; Bitonti and Mariotti 2023). Second, trust in political institutions is relevant in this regard: higher trust in political institutions is linked to a more positive view of lobbying. However, the higher level of trust in Swiss political institutions, as compared to Canada, does not translate into a more positive perception of lobbying in the country. This suggests that regulation may serve as the key determinant of public attitudes towards lobbying, rather than trust in institutions. The fact that Swiss respondents express a negative perception of lobbying despite trusting their institutions implies that they place more value on how well lobbying activities are regulated and controlled. It is also plausible that the lower trust in political parties in Switzerland could contribute to negative views of lobbying, even if it is high for the other political institutions. This relationship makes sense when considering that political parties often serve as gatekeepers in political processes (Easton 1953).

In Switzerland, where institutional trust is significantly higher, the public perception of lobbying remains cautious or negative. This reinforces the finding in the model that perceptions of regulatory adequacy are likely a stronger driver of lobbying attitudes than trust alone. In countries like Switzerland, even high institutional trust does not reduce public concerns about lobbying. Therefore, policymakers aiming to improve lobbying perceptions may need to focus on ensuring that lobbying regulations are perceived as adequate and transparent rather than relying on institutional trust as a mitigating factor. At the micro level, other variables are significant predictors of lobbying perception, such as gender, age, and the level of education.

On the macro level, the two countries differ in their political systems, exhibiting the typical characteristics of majoritarian (Canada) and consensual (Switzerland) democracies (Lijphart 1999). Interestingly, lobbying is perceived better in the majoritarian case than in the consensual one. A possible explanation may concern the channels of ‘voice’ and representation available to citizens and interest groups in the two cases. In a ‘winner takes all’ majoritarian system (Canada), lobbying may represent the easiest option for those wishing to access policymaking processes. In a consensual democracy (Switzerland) with multiple parties, a collegial executive branch including all major parties, and a very meaningful space reserved to direct democracy, citizens have countless channels of voice and representation at their disposal (Sciarini et al. 2015; Christiansen et al. 2018; Eichenberger 2020), thus they may seem lobbying as something unnecessary or prevalently used by corporate players. In terms of political culture and relations between interest groups and governmental actors, a more pluralist system such as the Canadian one may thus favour a better perception of lobbying than is the case in a country more typically characterized by a neo-corporatist tradition (Lijphart 1999, p. 177), in line with previous studies on northern European countries (Larsson 2007; Helgesson and Falasca 2017). Various institutional differences may be also relevant in this regard. In Canada’s Westminster system, policymaking is highly centralized, with the Prime Minister’s Office and the governing party exercising significant control over the legislative agenda (Bakvis and Wolinetz 2005). As a result, lobbying efforts primarily target cabinet ministers and executive officials, whose activities are subject to disclosure requirements under the Lobbying Act (2008) (Chari et al. 2019). The formalization of lobbying through regulatory oversight contributes to its greater legitimacy in the eyes of the public, aligning with our findings that Canadians perceive lobbying more positively. In contrast, Switzerland’s semi-direct democracy and consensual system distribute policymaking authority more broadly, involving multiple institutional actors and direct democratic instruments such as referenda and popular initiatives (Sciarini et al. 2015; Christiansen et al. 2018; Eichenberger 2020). As Swiss policymaking advances through these multiple channels, lobbying takes place in less formalized ways, remaining largely unregulated (Ammann 2024). Therefore, while Swiss institutions enjoy high levels of public trust, the lack of transparency in lobbying practices may reinforce scepticism about interest group influence, explaining why Swiss citizens are more likely to perceive lobbying as an avenue for undue influence rather than a legitimate policy input mechanism. This suggests that the perceived quality of lobbying regulation, rather than trust in institutions alone, is a decisive factor in shaping public attitudes towards lobbying, a finding that has important implications for the broader study of democratic accountability and interest group influence.

## Limitations and future research

A limitation of this study refers to a contextual factor that may be taken into account—and that we do not consider in the present study—and it is the portrayal of lobbying by the media. While some consonance might be assumed between the media representations and citizens’ perception of lobbying in a given country, the literature on this topic lacks comparative studies, being mostly based on analyses of single case studies developed in countries such as the United Kingdom or Sweden. Unfortunately, no studies have been undertaken for Canada and Switzerland on this subject.

Our findings come with another main limitation. While our study has the advantage of relying on a comparative analysis, it is based only on two cases. More comparative work on a higher number of countries, covering other meaningful cases such as the United States or less studied countries (for instance, Global South countries), should be undertaken in a more articulated comparative setting.

Future studies on this issue might then pursue these further analyses, refining our understanding of the public perception of lobbying among citizens and other important players of democratic political systems.

Other interesting directions for future research could point to analyses of trust in political parties separately from general trust in political institutions, and to the impact of satisfaction with current governments on both trust and perception of lobbying. A preliminary analysis shows that combined trust and satisfaction significantly influence perceptions of lobbying. In particular, the relationship between trust and lobbying perception could depend on government satisfaction levels.

## Appendix

This Appendix includes information on the final sample on which the analyses were conducted, the operationalization of the dependent and independent variables, and the distribution of their frequencies (See Tables 5, 6, 7, 8, 9, 10 and 11).

In addition, it includes a sensitivity analysis which supports the results of the multinomial logistic regression.

*Sampling weights* General population weights are applied to adjust the sample distribution to align with the known population proportions for gender, age, and linguistic regions in Canada and Switzerland. This ensures the data are representative of these key demographic variables.

## Sample description

**Table 5:** Characteristics of the final (weighted sample)

Socio-demographic characteristics		Canada		Switzerland		Total	
		*N*	% on total for CA	*N*	% on total for CH	*N*	%
Gender	Male	1951	49.4	811	49.1	2762	49.3
	Female	1975	50.1	819	49.6	2794	49.9
	Some other way	20	0.6	21	1.3	41	0.7
	Total	3946	100.0	1651	100.0	5597	100.0
Age	18–29	732	18.6	210	12.7	942	16.8
	30–44	983	24.9	497	30.1	1480	26.4
	45–64	1382	35.0	626	37.9	2008	35.9
	65 years and over	849	21.5	318	19.3	1167	20.9
	Total	3946	100.0	1651	100.0	5597	100.0
Level of education	Less than primary school	20	0.5	4	0.2	24	0.4
	Complete primary school	111	2.8	14	0.8	125	2.2
	Complete secondary school/high school	1087	27.5	67	4.1	1154	20.6
	Basic vocational education and training (post-secondary school, non-tertiary school) (e.g. Apprenticeship)	830	21.0	728	44.1	1558	27.8
	Short-cycle tertiary education (e.g. professional courses)	638	16.2	223	13.5	861	15.4
	University education obtained Bachelor’s degree or equivalent	893	22.6	297	18.0	1190	21.3
	University education, obtained Master’s degree or equivalent	286	7.2	264	16.0	550	9.8
	University education obtained Doctoral degree or equivalent	81	2.1	54	3.3%	135	2.4
	Total	3946	100.0	1651	100.0	5597	100.0

## Dependent variable

**Table 6:** Perception of lobbying—Original variable based on the question “Below you find two opposite sentences on lobbying and democracy. Mark your position on a scale from 1 to 7, where 1 represents complete agreement with the sentence on the left, and 7 represents complete agreement with the sentence on the right”. Number of responses by type of perception and related valid %

	Canada		Switzerland		Total	
	*N*	%	*N*	%	*N*	%
1—Lobbying is always a danger to democracy, as it represents a form of corruption	379	9.6	290	17.6	669	12.0
2	321	8.1	300	18.2	621	11.1
3	433	11.0	370	22.4	803	14.3
4	1265	32.1	491	29.7	1756	31.4
5	754	19.1	153	9.3	907	16.2
6	444	11.3	34	2.1	478	8.5
7—Lobbying is good for democracy, as it helps policymakers gather information and make better decisions	350	8.9	13	0.8	363	6.5
Total	3946	100.0	1651	100.0	5597	100.0

The original variable was recoded into three categories for the bivariate and multivariate analyses, showing negative, positive, and neutral perceptions.

**Table 7:** Perception of lobbying recoded—Number of responses by type of perception and related valid %

Original values	Recoded values and labels	Canada		Switzerland		Total	
		*N*	%	*N*	%	*N*	%
1, 2, 3	1—Lobbying is a danger to democracy (Negative)	1133	28.7	960	58.1	2093	37.4
4	2—Neither a danger nor beneficial (Neutral)	1265	32.1	491	29.7	1756	31.4
5, 6, 7	3—Lobbying is beneficial for democracy (Positive)	1548	39.2	200	12.1	1748	31.2
Valid total	3946	100	1651	100	5597	100	

## Independent variables

1. Citizens’ perception of the appropriateness of the lobbying regulation in their country
  This independent variable is measured by asking, “Do you think lobbying is properly regulated in your country?”. The response categories entail “Yes”, “No”, and “I don’t know”. The following table shows the distribution of this variable by country.

**Table 8:** Citizens’ perception of the appropriateness of the lobbying regulation in their country Number of responses and valid % by country

		Country		Total
		Canada	Switzerland	
No	*N*	1525	896	2421
	%	38.6	54.3	43.3
Yes	*N*	728	362	1090
	%	18.4	21.9	19.5
I don’t know	*N*	1693	392	2085
	%	42.9	23.8	37.3
Total	*N*	3946	1650	5596
	%	100.0	100.0	100.0

2. Citizens’ trust in political institutions
  The main independent variable concerns citizens’ trust in political institutions. This concept is measured as the arithmetic mean of six different variables on the level of trust in different political institutions (i.e. the Parliament/General Assembly, local representatives, provincial/cantonal representatives, political parties, the party in power/Federal Council, the Prime Minister/President). These six variables were measured through a seven-item Likert scale asking ““How much trust do you have in the following political institution?”.

**Table 9:** Citizens’ trust in political institutions. Number of responses and valid % by country

			Country		Total
			Canada	Switzerland	
Index of trust in political institutions	low trust (0.0–0.34)	*N*	2867	860	3727
		%	74.8	53.8	68.6
	Medium trust (0.34–0.66)	*N*	223	119	342
		%	5.8	7.4	6.3
	High trust (0.66–1.0)	*N*	745	620	1365
		%	19.4	38.8	25.1
Total		*N*	3835	1599	5434
		%	100.0	100.0	100.0

Reliability analysis was conducted for the 'Trust in Political Institutions' scale, yielding a Cronbach's Alpha of 0.903, indicating excellent internal consistency across the six items.

**Table 10:** Reliability analysis of the Index of Trust in Political Institutions. Cronbach’s Alpha

Scale	Cronbach's Alpha	No. of items	Mean	Std. Deviation
Trust in political institutions	0.903	6	1.747	2.226

Notes on the regression model: Several regression methods and models have been tried to confirm the significance of the final choice (i.e. binary logistic regression, original regression PLUM, linear regression, and multinomial logistic regression). All regressions confirm the results of the chosen multinomial regression model.

Table 11 below, includes the linear regression results, which can be used as sensitivity analysis and to demonstrate the lack of collinearity among the independent variables.

**Table 11:** Results of linear regression

Variable	*B*	SE B	*β*	*t*	*p*	Tolerance	VIF
(Constant)	4.980	0.139	–	35.894	< 0.001	–	–
Country	−1.209	0.049	−0.329	−24.596	< 0.001	0.879	1.138
Trust in Parliament/General Assembly (q2301)	0.115	0.073	0.032	1.578	0.115	0.383	2.608
Trust in Local Representatives (q2302)	0.175	0.062	0.049	2.801	0.005	0.509	1.966
Trust in Provincial/Cantonal Representatives (q2303)	0.094	0.071	0.026	1.326	0.185	0.415	2.411
Trust in Political Parties (q2304)	0.329	0.070	0.079	4.675	< 0.001	0.545	1.835
Trust in Federal Council Power (q2305)	0.212	0.080	0.059	2.638	0.008	0.314	3.188
Trust in Prime Minister/President (q2306)	0.224	0.077	0.063	2.906	0.004	0.336	2.972
Left-Oriented (q29)	−0.346	0.044	−0.100	−7.777	< 0.001	0.943	1.060
University	−0.067	0.044	−0.020	−1.529	0.126	0.936	1.069
Perception of Lobbying Regulation	0.004	0.000	0.118	8.797	< 0.001	0.879	1.138
Frequency of Following National Politics (q25)	−0.061	0.021	−0.043	−2.948	0.003	0.734	1.363
News Consumption Over Last Three Years (q27)	0.063	0.034	0.025	1.855	0.064	0.894	1.119
Voting in the Last Federal Election (q32)	0.003	0.001	0.042	3.275	0.001	0.962	1.040
Age	−0.004	0.001	−0.039	−2.804	0.005	0.822	1.217
Gender (REC)	0.259	0.042	0.079	6.137	< 0.001	0.937	1.067

Dependent variable: perception of lobbying (1–7 Likert scale). *B* unstandardized coefficients, SE B standard error of B, *β* tandardised coefficients, *p* values are two-tailed

Several multinomial regression models with different regressors were tested. Below, we provide details on why these additional regressors were not included in the final model.

- The quality of public service (coefficient not significant)
- The level of satisfaction with the current government (significant, but it severely decreased the stability, in terms of sparse cells, of the model and only increased the explained variance by 0.4%)
- Age (significant, but it severely decreased the stability of the model and only increased the explained variance by 0.3%)
- Interaction trust&satisfaction (significant, but it severely decreased the stability of the model and only increased the explained variance by 0.2%)
- Interaction age&trust (significant, but it severely decreased the stability of the model and only increased the explained variance by 0.2%)

Trust, regulations, and gender were the most significant and strongest predictors across all combinations.

The final model chosen and included in the manuscript shows the best trade-off between the significance of predictors (Likelihood Ratio Tests for each predictor show highly significant results (*p* < 0.001)), model fit/explained variance (Nagelkerke: 0.318; 32%), stability (8.6% zero cells) and interpretability of results. We prioritized model stability over explained variance. Model stability is often more crucial than maximizing explained variance in social science research. Stable models help ensure that findings are robust, interpretable, and replicable across different samples or contexts, which is vital when studying complex social phenomena.

## Footnotes

1 It must be noted that trust can be considered from different perspectives. On the one hand, it can be a result of more robust lobbying regulations, as a dependent variable (Crepaz and Arikan 2024). On the other hand, it can be considered as an independent variable affecting the public perception of lobbying. In this article, we focus on this second perspective.

2 While Switzerland does not have a specific law on lobbying, its regulatory framework may be portrayed in a more nuanced way, also depending on the definition of lobbying regulation one adopts. See the discussion later in this section.

3 Zero cells indicate combinations of variable levels where there are no observations, which can sometimes weaken the model.
